# Influence of Resilience on Burnout Syndrome of Faculty Professors

**DOI:** 10.3390/ijerph19020910

**Published:** 2022-01-14

**Authors:** Blanca Rosa García-Rivera, Ignacio Alejandro Mendoza-Martínez, Jorge Luis García-Alcaraz, Jesús Everardo Olguín-Tiznado, Claudia Camargo Wilson, Mónica Fernanda Araníbar, Pedro García-Alcaraz

**Affiliations:** 1 Faculty of Administrative and Social Sciences, Universidad Autónoma de Baja California, Valle Dorado, Ensenada 22890, BC, Mexico; maranibar@uabc.edu.mx; 2 Department of Postgraduate Studies, Universidad Anáhuac, Anáhuac 01840, DF, Mexico; alejandro.mendozam@anahuac.mx; 3 Department of Industrial Engineering and Manufacturing, Autonomous University of Ciudad Juarez, Ciudad Juarez 32310, CHI, Mexico; 4 Faculty of Engineering, Architecture and Design, Universidad Autonoma de Baja California, Ensenada 22860, BC, Mexico; jeol79@uabc.edu.mx (J.E.O.-T.); ccamargo@uabc.edu.mx (C.C.W.); 5 Instituto Tecnológico de Colima, Villa de Álvarez 28976, CP, Mexico; alcarazgarciapedro@yahoo.com.mx

**Keywords:** burnout syndrome, COVID-19 pandemic, resilience, work-related exhaustion, professors

## Abstract

This research aims to describe the relationship between resilience and burnout facing COVID-19 pandemics. The sample was *n* = 831 lecturers and professors of a Mexican public university. This study is a quantitative, non-experimental, cross-sectional, explanatory, and ex post facto research using Structural Equations Modeling with latent variables under the partial least square’s method technique. We used the CD-RISC-25 and SBI questionnaires to measure resilience and burnout, respectively. Structural Equations Modeling (SEM–PLS) allowed the visualization of the exogenous variable (resilience) in endogenous variables (dimensions of SBI burnout: E9 guilt, E7 emotional exhaustion, E8 indolence, and E6 work illusion). To this day, there are very few previous studies that jointly analyze in Mexico the characteristics of resilience and burnout in the face of the COVID-19 pandemic. Findings show that resources availability has the strongest correlation with accomplishment in teaching, followed by cynicism and emotional exhaustion. These results have important professional implications.

## 1. Introduction

### 1.1. Burnout Syndrome

Burnout, also known as professional exhaustion, is a process of chronic stress that evolves if not prevented and affects people psychologically [1]. Exposure to high amounts of stress over a long period plays an important role in developing the syndrome [2]. Freudenberger [3], a pioneer in the early 1970′s noticed that the syndrome caused high emotional weariness, high depersonalization, and poor professional fulfillment [4,5].

Over the last year, burnout has become a critical issue due to social and personal problems associated with the COVID-19 Pandemic. It is affecting millions of humans that work in service professions. Burnout has been related to the new normality associated with distance work and isolation, attitude and behavioral factors of students, health issues, uncertainty, and poor working conditions [6,7,8,9]. The COVID-19 Pandemic affected faculty members all across the world. It is worth nothing that acquiring good data about COVID-19 has been a difficult task. The majority of COVID-19 data released has been of low quality [10] every day. COVID-19 has been a challenge for faculty lecturers and professors because it increases burnout due to isolation, stress and poor working conditions. In European countries such as Italy, Germany, the United Kingdom, and Belgium, fatality rates were higher than 10% of the population. Other nations, such as Thailand and Japan, had a minor mortality rate of less than 2% [11]. Latin America and Mexico, however, showed higher rates during 2020 that reached more than 13%. Hence, results of this study cannot be generalized globally.

The World Health Organization [12,13], has classified burnout as an illness; a syndrome caused by prolonged occupational stress that has not been effectively controlled. In fact, it develops emotional tiredness, which is thought to affect up to 50% of employees who work in public and service professions [14]. Mental illness may develop due to factors related to occupational stress, affecting workers in all biological, social, and psychological areas.

Currently, this topic has gained popularity in Mexico due to the Mexican standard NOM-STPS-035-2018, which recently obliges organizations to monitor their workers` mental and emotional states. Burnout syndrome has been recognized as a psychosocial risk that threatens public workers’ mental and emotional health. It has been described as a gradual process that arises within professionals who continually work with people, therefore, suffering emotional exhaustion [15,16,17,18]. Previous research describes burnout as a syndrome resulting from chronic workplace stress that has not been successfully managed [19]. Maslach, one of the main experts in burnout, describes it as a gradual process that occurs after chronic stress, represented by three dimensions: depersonalization, low personal accomplishment, and emotional exhaustion [14,15,16]. She also notes that this syndrome has a social connotation since it arises within workers who continuously work in service professions, including professors, police officers, psychologists, social workers, nurses, physicians, care workers, among others [20]. Previously, [21] found that burnout was related to physical, emotional, and mental exhaustion where a lack of interest in work, low level of accomplishment, and dehumanization were observed.

Burnout can be caused by job demands and lack of resources. Previous research has shown that stress represents a mismatch between job demands and resources availability [22]. When there is a balance of job demands with job resources, professors can successfully meet their job demands. [23,24], and the unbalance between them may eventually lead to professor burnout and attrition [25]. As a result, one of the most impacted sectors by the burnout syndrome is education [26].

In this sense, a very high percentage of burnout is observed in faculty professors, widely reported in several studies [27,28,29]. however, there is a gap in research concerning the influence of resilience and the development of burnout in the new normality. Due to the importance of these findings, we consider that this research will bring new light to the situation.

The appearance of burnout in professors has been related to their intense and extended interaction with students who demand attention and a great investment of time, mental and emotional efforts. Hence, dealing with lack of support and institutional recognition, low job satisfaction, inadequate wages, devaluation of teaching work, poor resources and a feeling of having a job with a never-ending list of tasks, brings, as a result, indifference and feelings of failure, incompetence, disillusion and burnout arises slowly [30,31,32,33,34,35,36,37,38,39,40,41,42,43].

Since the presence of COVID-19 in 2020, global measures of protection and isolation forced universities to declare quarantine. Professors faced the compulsory closure of institutions and the continuity of work from home. For many professors, online teaching represented a considerable challenge: they found themselves, in a lockdown, having all family members confined in the same limited spaces, sharing equipment, and with insufficient resources to perform their tasks. In addition, feelings of anxiety, distress, fear, and depression emerged after an extended period of social isolation [44].

Moreover, burnout has been defined as a psychosocial risk that evolves in work settings, and it is tied to stress and behaviors related to professional disillusionment, los of work excitement, affective deterioration, emotional exhaustion, the appearance of negative attitudes and behaviors, and cold, indifferent, distant, cynical, and insensitive behaviors. Different models such as the three-dimensional model of the MBI-HSS [45], the model of Edelwich and Brodsky [46], the model of Price and Murphy [47], and the SBI model of Gil-Monte [48], among others [49,50] have demonstrated the connection of these behaviors with lack of coping resources and high emotional demands faced by professors and care workers. The model of Gil Monte is characterized by cognitive impairment, poor work excitement, and disillusionment. Feelings of low personal accomplishment and physical and work-related exhaustion are shown by professors, followed by negative attitudes and behaviors towards students, administrative and teaching staff with cynical, indifferent, cold, and distant behaviors, sarcasm, passive aggression, depersonalization, and lack of sensitivity to situations that require empathy, experiencing feelings of guilt when performing these attitudes [51,52,53,54,55,56].

Research has shown that professors who perceive a lack of support from colleagues and supervisors have diminished self-efficacy beliefs, predicting higher levels of burnout [57]; poor working conditions were found to be predictors of emotional exhaustion and low personal accomplishment [58]. Burnout increases adverse reactions to students. Burnout increases the incidence of mental health problems [59]. Professors’ burnout is related to psychosomatic symptoms, exhaustion, insomnia, ulcer, shoulder and neck pain, and increased family conflicts.

### 1.2. Resilience

The ability to adapt to adversity is referred to as resilience [60]. Positive emotions (happiness, optimism, self-esteem, and assertiveness) will be critical in the face of these challenging situations [61]. As a result, the word “resilience” refers to the protective features that humans build through time. In other words, this construct is not a concept that is inherited and passed down from generation to generation, but rather a term that is acquired and developed throughout life and via adverse experiences and emotional control that individuals have over themselves [62].

Resilience is understood as an adaptation to high stress or trauma. Individuals with high resilience develop protective factors and resources to adapt to and emerge from adversity [63,64]. Research has found that resilience has been defined as “the capacity to recover and maintain an adaptive sanity after being abandoned or the initial capacity to start a stressful event” [65]. It is also defined as “the human capacity of confronting, overcoming and being strengthened or transformed by the experiences of adversity” [66]. Due to COVID-19 in the world, the current pandemic scenario is a challenge and a threat to the human resistance process in every way. Resilience in professors is described as an eight-dimension construct formed by confronting mechanisms, autonomy, self-esteem, awareness, responsibility, hope, sociability, tolerance, and frustration. Each of these is a base of the resilient support on the human being towards adverse situations [67].

Research shows that resilience implies protective factors as being easy-going, a good sense of humor, positive relationships, a strong sense of self-worth, feeling a sense of control over work and personal circumstances, feeling effective at work, relationships, recreation, and approachable, informal social network, above-average social intelligence, being flexible and able to delay personal gratification, believing in one’s self-efficacy, having an internal locus of control, the ability to problem-solve, being able to trust in others, having hope for the future, the capacity for critical thinking, and high expectations of oneself [68,69,70,71,72]. Research has shown that resilience is an essential mechanism for well-being [73]. It has been described as “an individual’s process in surviving in the face of adversity or other conditions that cause the individual to feel depressed, miserable, or traumatized” [74]. Resilience is the ability to be able to respond healthy and productively while in pressure situations. It develops own capabilities to overcome and adapt to difficult situations, to the point that resilience can determine success or failure in life [75,76].

However, there is a gap in research concerning the influence of resilience and the development of burnout in the new normality. Due to the importance of these findings, we consider that this research will bring new light to the situation.

Then, a research question emerged: How does resilience influence the work-related burnout of faculty professors?

### 1.3. Objective and Hypotheses

#### 1.3.1. Objective

This paper analyzed the influence of resilience in burnout built by professors considering the contextual resources. The objective was to determine the influence of resilience on burnout dimensions in faculty professors under a Structural Equation Modeling of latent variables SEM-PLS. In contrast with previous research studying the relationship of burnout and its dimensions with other variables is essential to describe the new normality parameters related to resilience and the burnout syndrome.

#### 1.3.2. Hypotheses

The hypotheses proposed, include the burnout syndrome dimensions according to Gil-Monte’s Model (guilt, mental exhaustion, indolence and work excitement).

**Hypothesis** **1 (H1).** *E14 resilience has a significant inverse influence on E9 guilt.*

Guilt is a dimension of burnout in Gil-Monte’s Model. He defines it as the development of feelings of guilt from negative attitudes and behavior evolving on the job, especially towards students and people with whom the professor establishes work relationships. Previous research shows significant differences related to gender, significantly higher scores in women than in men [77]. Most studies show that women present higher scores on the guilt scale than men [78]. Guilt is a strong predictor of burnout [79,80]. Previous research has shown that feelings of guilt have been identified as one of the most destructive factors in burnout [81].

Guilt is frequently related to negative thoughts about oneself and the negative and cynical way we have treated others. Professors most of the time underestimate stressful situations that lead them to personality malfunction, blaming themselves for performing their job inadequately. In this process, Professors develop a sense of failure and a loss of self-esteem [82]. There are feelings of becoming cold and dehumanized. This cycle then makes them reaffirm their commitment, responsibility, and care for others [83], and finally, as a result, higher levels of burnout are developed.

Research shows that resilience diminishes guilt, studies show that high levels of resilience predict lower levels of guilt; resilience showed a negative correlation with guilt in most studies reported, resilience helps professors to cope with the stressors they experience and reduce feelings of burnout and guilt [84,85,86].

**Hypothesis** **2 (H2).** *E14 resilience has a significant inverse influence on E7 mental exhaustion.*

Mental exhaustion is defined by Gil-Monte as the appearance of emotional and physical exhaustion linked to the work carried out. Previous research shows significant differences related to gender, significantly higher scores in men than in women. Most studies show that men present higher scores in the exhaustion dimension than women [46,47,87]. Exhaustion is a strong predictor of burnout. The mental exhaustion dimension is described as wearing out, losing energy, debilitation, depletion, and fatigue [47,87]. On the other hand, resilience is a vital factor that diminishes professional exhaustion in professors [46,87]. Academic resilience is of great importance for professors. According to [88,89,90], resilient professors can change a challenging environment into a source of motivation by keeping hope, setting up goals, and teaching their students problem-solving skills. Research has shown that professors might also have high subjective well-being in facing online learning challenges during the COVID-19 pandemic [91]. Professors with higher resilience feel more optimistic and develop beliefs of a better future.

**Hypothesis** **3 (H3).** *E14 resilience has a significant inverse influence on E8 indolence.*

Indolence is defined as a process that leads to the appearance of negative attitudes of indifference and cynicism when dealing with students; persons having high rates of indolence are insensible and do not worry about others´ problems [92]. It is also described as negative attitudes, irritability, withdrawal, and loss of idealism. Professors who suffer high levels of indolence have a low service rate and produce a higher rate of complaints and less motivation in students. On the contrary, Evidence also indicates that resilience is essential in managing indolence in teachers [93,94].

**Hypothesis** **4 (H4).** *E14 resilience has a significant direct influence on E6 work excitement.*

Enthusiasm towards the job, also known as work excitement, is explained by Gil-Monte as the ambition to accomplish professional goals because they represent a source of personal accomplishment and a personal source of pleasure. Individuals perceive their job as attractive and to achieve goals is a source of personal accomplishment. The items in this dimension are in a positive direction. Lower rates of this dimension indicate higher levels of burnout. In addition, resilience has been described as a dynamic construct that helps professors cope with hardship and fuel them with positive energy, including effectiveness, passion, life satisfaction, perseverance, and other variables associated that lead to higher enthusiasm towards the job [95,96,97].

In light of the current literature regarding resilience and burnout subscales, we predict as follows in Table 1 four hypotheses:

Four hypotheses are presented in this table. Three of them were developed inverse, and they refer to the influence that resilience has on faculty professors against guilt, mental exhaustion, indolence, and work excitement; dimensions that measure burnout in the SBI Model.

## 2. Methods

### 2.1. Survey Design

**Figure 1:**
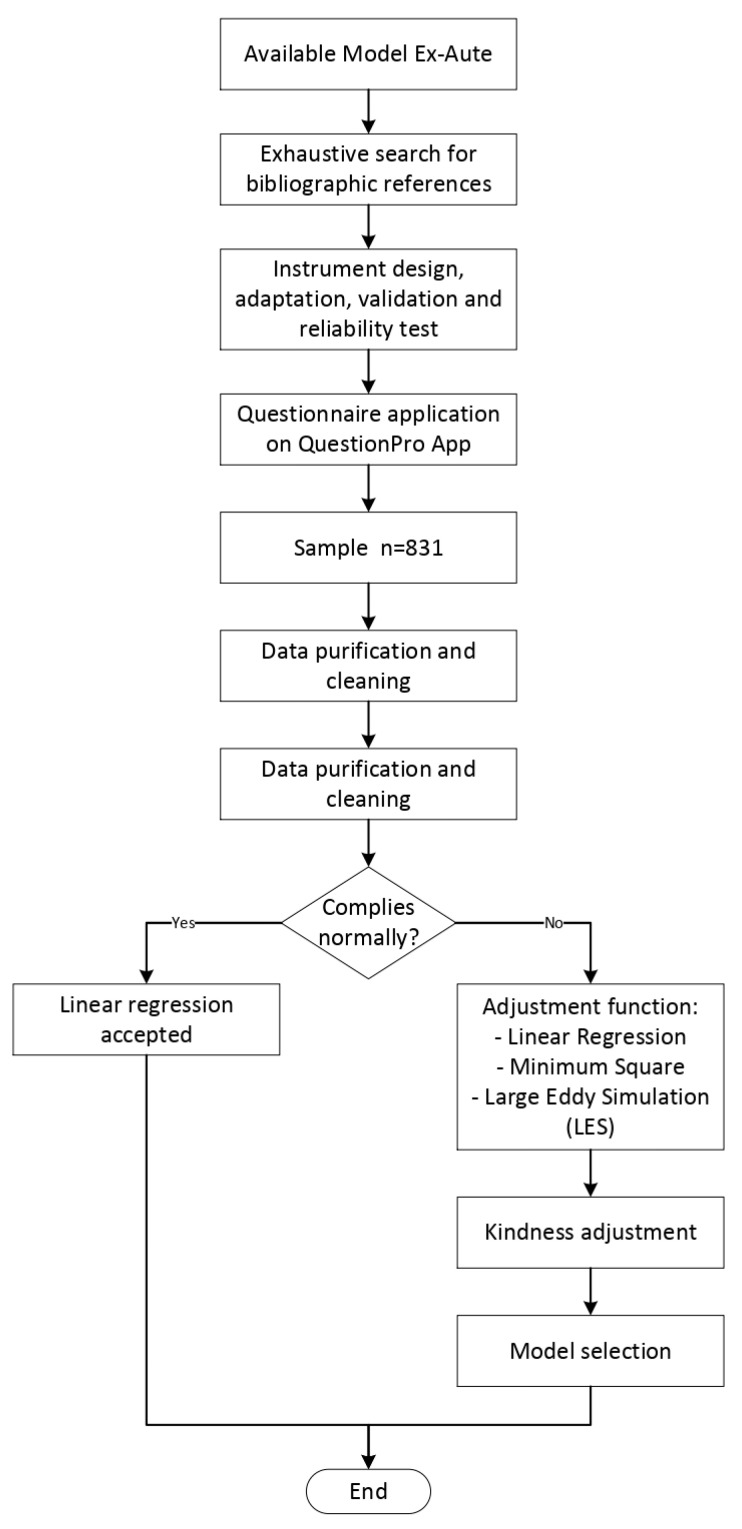
Work Flow. Source: Own elaboration.

This research aim was to find the relation between professors’ resilience and burnout. Following, in Figure 1 we can see a work flow of the investigation.

As indicated in Figure 1, after exhaustive search and questionnaire adaptation and design, we used the SBI questionnaire to measure burnout designed by Gil-Monte and the CD-RISC-25 questionnaire to measure resilience [98], despite the fact that resilience was constructed as a single-dimensional concept for this study. The final questionnaire has three sections; the first part includes demographic profile questions of the participants; burnout was measured using the SBI [99]. This instrument is comprised of 20 items distributed into four: enthusiasm towards the job also known as work excitement, (5 items); psychological exhaustion (4 items); indolence (6 items); and guilt (5 items). The items are evaluated on a 5-point frequency scale from 0 “Never” to 4 “Very frequently: every day.” To measure resilience, we used the CD-RISC-25 scale. This instrument comprises 25 items distributed into five factors: The first factor (8 items) reflects the notion of personal competence, high standards, and tenacity. The second factor (7 items) has to do with trust in one’s intuition, tolerance of negative affect, and the strengthening effects of stress. The third factor (5 items) reflects positive acceptance of change and secure relationships. The fourth factor (3 items) reflects control. The fifth factor (2 items) reflects spiritual influences. The questionnaire uses a Likert scale of 7 points to evaluate every item indicating agreement with the statements. These ratings result in between 0–100, and higher scores indicate higher resilience [100].

### 2.2. Survey Application

The study is built on a survey-based design. This survey was conducted in 2020 when all professors were engaged in teaching remotely online and isolated. The survey was anonymous and required 15 min to complete. To complete the questionnaire, it was required to answer all the previous questions.

The questionnaire was electronically uploaded to a platform called QUESTION-PRO. It was reviewed and approved by the University Research Ethics Board. Once approved, the questionnaire was sent in an institutional email to all current professors, inviting them to complete it. The survey was anonymous. The population of university professors is N = 4000. Of the total, 831 answers were received, representing 20.77%.

### 2.3. Data Capture and Debugging

The information obtained from questionnaires was uploaded into an edited and analyzed database in the Statistical Package for the Social Sciences (IBM SPSS) version 25 for Windows. Each row indicated a response or case, and the columns indicated the items for every dimension analyzed.

The database went thru the following process:

1. To identify extreme values, answers were standardized, and any value above four was considered extreme, then replaced by the median.
2. To identify any participant with no commitment, the standard deviation of every case below 0.5 indicated that similar values were always chosen. As a result, that case was dismissed from the analysis.
3. There were no missing values since the electronic questionnaire was conditioned to answer all the questions to complete and send.

### 2.4. Descriptive Analysis

To know the item’s path and its level of impact, we obtained the following parameters: the median of each item as central path value. Higher values indicated a more substantial presence in the professors’ group, and lower values indicated a weaker presence—standard Deviation as disperse measurement. Higher values indicate higher dispersion of data and common consensus of the participant concerning the item’s values. Lower values indicated lower dispersion of data and higher consensus between the participants.

Several parameters were obtained for dimensions validation before integrating them into the structural equation Modeling. For example:

Cronbach’s alpha reliability, Composite reliability, and Rho A coefficients are used to measure internal consistency. This value is obtained iteratively because by eliminating some items, the value increases.

The Average Variance Extracted (AVE) measures the variance extracted from every dimension and indicates discriminant validity.

The Discriminant coefficients (Square root of AVE) are used to confirm the instrument’s validity at the optimal level, affirming that the instruments measure what they intend to do so.

### 2.5. Structural Equation Modeling

**Figure 2:**
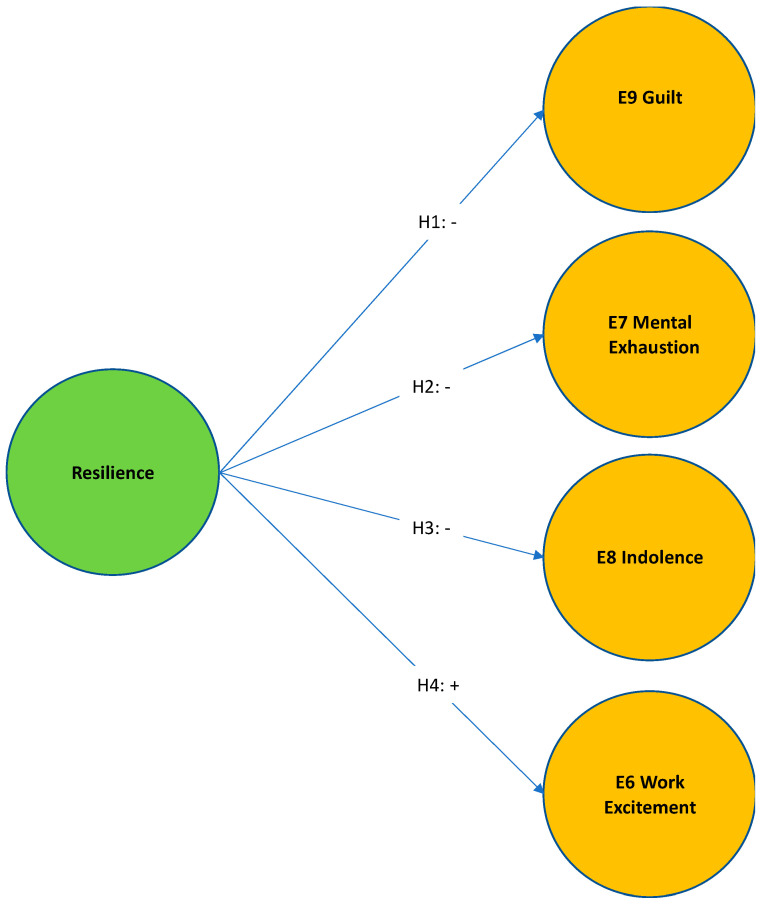
Ex-Ante Model. Source: Own elaboration.

Once the items were validated, they were integrated into a Structural Equation Model. Following, we propose the Ex-ante Model in Figure 2.

As shown in Figure 1, direct effects are observed between the variables; hence we decided to use β to analyze the independent latent variable and the dependent one, which is associated with a *p*-value to find the significance of it, where the null hypothesis is H0: β = 0, versus the alternative Hypothesis 1: β ≠ 0. This way, if found that β = 0, we can conclude that there is no relation between the analyzed variables, but if β ≠ 0, then we conclude that there is a relation between them.

## 3. Results

### 3.1. Sample Statistics

The instruments used for data collection include a series of socio-demographic variables such as gender, marital status, age, level of schooling, type of worker, seniority.

Following, we present Table 2 with the demographics of the participants/Faculty members:

As noticed in Table 2, Research subjects who collaborated in this project were faculty professors at a Mexican public university located in Baja California, *n* = 831. Their demographic and organizational data show that most of them were married, middle aged, adjunct professors, not preparing for postgraduate studies, with more than one job and 10 or more years for retirement.

### 3.2. Descriptive Analysis

Following, in Table 3, we present differences between men and women:

As noticed in Table 3, there are no significant differences in E6 work excitement W = M, for E7 mental exhaustion, there are significant differences W > M; for E8 indolence, there are significant differences W > M; for E9 guilt, there are significant differences W < M; for E14_resilience, there are significant differences W < M.

### 3.3. Latent Variable Validation

In order to assess the validity of the instruments, the use of the Confirmatory Factor Analysis was necessary, complemented with the analysis of the Average Variance Extracted (AVE) and the Discriminant Validity. For the analysis of the reliability of the instrument, the coefficients of Cronbach’s alpha, Rho_A, and Composite Reliability (CR) were obtained. The Pearson product-moment coefficients were calculated. The structural modeling (SEM–PLS) was developed under the theoretical foundations and the reflective method. All the constructs had a factorial load higher than 0.70, which makes them valid for each variable (please see Appendix A).

### 3.4. Structural Equation Model

The Structural Equations Modeling (SEM–PLS) allowed the visualization of the exogenous variable (Resilience) in endogenous variables (E9 guilt, E7 mental exhaustion, E8 indolence and E6 work excitement) with the corresponding items, thus evaluating jointly these hypotheses.

The Pearson product-moment correlation coefficients allowed the observation of significant positive correlations between all the analyzed subscales. Significant inverse correlations were obtained between resilience and E9_work excitement with all the other subscales of burnout: E7_mental exhaustion, E8_indolence and E9_guilt (a significant direct correlation existing between these ones) confirming the theory.

Significant direct correlations were reported between E7_mental exhaustion and E8_indolence (r = 0.987) and E9_guilt (r = 0.244); whereas significant inverse correlations were observed between E6_work excitement and E7_mental exhaustion (r = 0.269), between E6_work excitement and E8_indolence (r = −0.289) and E9_guilt (r = −0.175).

A significant direct correlation was reported between E14_resilience and E6_work excitement (r = 0.450); whereas with all the other subscales of work-related exhaustion or burnout were significant inverse: E14_resilience with E7_mental exhaustion (r = −0.316), E8_indolence (r = 0.332) and E9_guilt (−0.234).

E14_resilience directly and significantly influenced from its standardized beta coefficient in E6_work excitement (0.488) and explained approximately 23% of its variance based on its R squared; while for the other subscales of burnout, its influence was significant inverse as follows: with E9_guilt (−0.238) and it explained approximately less than 1% of its variance based on its R squared; in the case of E7_mental exhaustion (−0.320) and explained approximately 10% of its variance based on its R square; in the case of E8_indolence (−0.279) and explained approximately less than 1% of its variance based on its R squared.

Results of the Descriptive statistics, Reliability, Validity and Correlations between the subscales of the Structural Equations Modeling of second-order trajectories with latent variables are displayed in Table 4.

In order to analyze the significant differences between resilience and the subscales of burnout, some statistical tests of mean differences were performed with the Student’s *t*-Test and the Variance Analysis.

Understanding the multivariate nature of the hypotheses and the research question, it was necessary to use Structural Equation Modeling with latent variables (SEM-PLS).

### 3.5. Hypotheses Test Results

The influence of resilience is explained as follows:

  Hypothesis 1: E14 resilience had an inverse significant influence on E9 guilt. A standardized beta coefficient of −0.238, explaining approximately 1% of its variance. We could observe that E14 resilience had very little influence in E9 guilt, even thought it was significant. We can affirm that “a higher E14 resilience reduces very little the amount of E9 guilt”. However, it is important to continue the study of the inverse relationship in later research to observe its significance level and influence or impact on E9 guilt.  Hypothesis 2: resilience had an inverse significant influence on E7 mental exhaustion, A standardized beta coefficient of −0.279, explaining approximately 1% of its variance. Thus, we can affirm that “a higher E14 resilience reduces E7 mental exhaustion”  Hypothesis 3: resilience had an inverse significant influence on E8 indolence, A standardized beta coefficient of −0.32, explaining approximately 10% of its variance. Thus, we can affirm that “a higher E14 resilience reduces E8 indolence”.  Hypothesis 4: resilience had a direct significant influence on E6 work excitement, A standardized beta coefficient of −0.488, explaining approximately 24% of its variance. Thus, we can affirm that “a higher E14 resilience increases E8 work excitement”.

**Figure 3:**
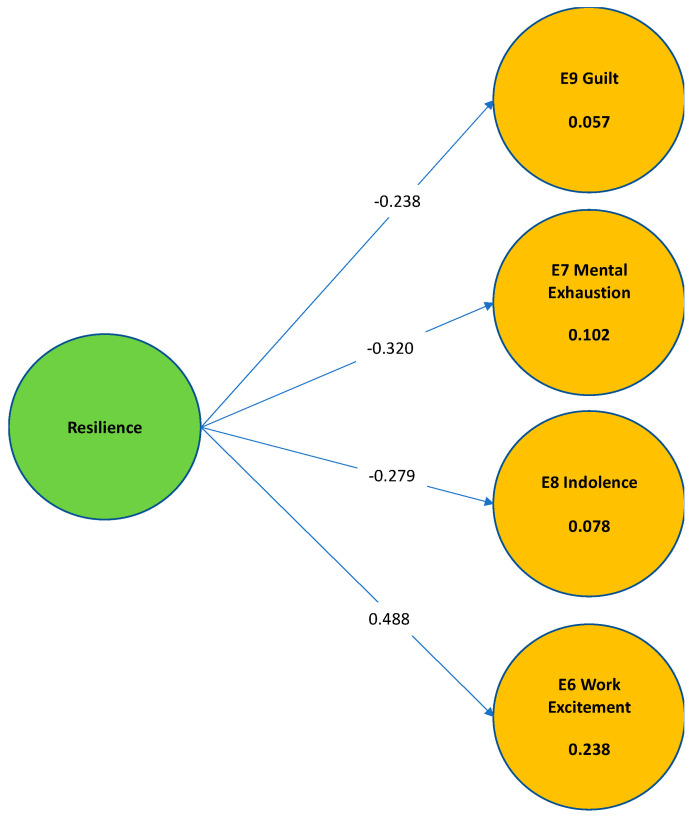
Ex-Post Model of structural equations of the hypothesis test. Source: Own elaboration.

Following, we present Figure 3 showing the Ex-Post Model:

In order to review the model, the bootstrapping was necessary again and it is displayed in Table 5.

As noticed in Table 3, we ran the SEM again with bootstrapping (using only 500 samples) to test that the results had a statistical significance. Results from bootstrapping were satisfactory in every hypothesis, with a value of *p* < 0.05 for all of them.

Finally, considering the hypotheses test results, we can observe the following findings in Table 6.

These findings about the influence and impact of burnout dimensions will be able to determine new action lines for psychological intervention in professors and lecturers, giving higher attention to resilience and work excitement to be able to diminish burnout factors as guilt, indolence and mental exhaustion.

## 4. Discussion

By Modeling a structural equation system, we could analyze the relationship between resilience and burnout; this study evaluated and interpreted the relationship between burn-out and resilience during the first phase of COVID-19. This study’s response rate reflects professors’ high interest in burnout during pandemics. In contrast to previous studies, a high percentage of professors suffered from this syndrome due to this experience.

To date, there has been a growing interest in online education, and this study is a response to that. The study was prompted by several factors, including COVID-19′s impact on society, mental and physical health issues, and resilience, that should be studied further. This study’s unique focus on the role of faculty professors in e-learning, resilience, and burnout is a notable feature of this research. In addition, the discipline is necessary because there is little evidence on the analysis of professional stressor factors linked to faculty professors. Health and teaching professionalization literature will benefit from the findings of this study.

This finding is crucial since earlier research has shown that online environments are innately isolating. Following the COVID-19 disruption, we discovered that isolation enhanced burnout and that resilience was important. It’s worth mentioning that professors and lecturers from the faculty of medicine who serve as physicians in local hospitals have a higher psychological influence on the COVID-19 pandemic due to their contextual experiences. A recent poll discovered that respondents in Europe had a moderate amount of emotional tiredness, a low level of depersonalization, and a high level of personal success [101]. These findings, when combined with the majority of physicians’ good financial effect and increased workload, create an atmosphere with a mixed bag of positive and bad repercussions. This outcome is most likely due to the perseverance of junior and middle-grade physicians, who reported high levels of personal success despite increased workload and severe pandemic impacts, it would be of importance to compare with Mexican and Latin American doctors these findings in future research.

The e-learning modality, in particular, has a high degree of flexibility and adaptability, which may explain in the future if burnout and psychological risks factors increase and then decrease after the adaptation is complete to new technologies and new paradigms in e-education. In addition, the sense of belonging to the teaching community is another exciting and essential part since, in the first period of isolation (2020), that sense was not reinforced because the role of distance educators differs from that of regular educators.

The purpose of this research was to describe the influence that resilience has on burnout and whether it is more significant in any of the subscales of burnout. As observed in this research, resilience itself does not explain burnout, and its influence on most burnout subscales is meager. However, it is observed that in the work excitement subscale, burnout is explained by more than 23%.

The current research findings offer some support for the SBI and CD-RISK Models [102,103]. Both [46,47,78,79] and [49,64,92,96] had similar results, and our research showed that during the pandemic, specific patterns emerged. Specifically, in the hypotheses examined, results followed the predicted pathway [90,92]. in previous research, they were treated as subsets of latent variables [93,94].

The findings confirmed the link between academic resilience and burnout by demonstrating that they are negatively associated in our sample. Our findings are consistent with prior research, which found that professors with stronger academic resilience were better able to avoid burnout than their peers at university [25,26]. In view researchers’ notion of academic resilience [29], this fact might be interpreted in a variety of ways. Academic resilience, such as school burnout, is defined by the authors as the ability to endure recurrent and overwhelming educational adversity and maladjustment. Furthermore, academically resilient professors are more enthusiastic and passionate about their students and class than their less resilient counterparts, as evidenced by prior research (e.g., [33,38]).

## 5. Conclusions

The current study, similar to previous research, emphasizes the importance of personal attributes such as resilience in protecting faculty members against extreme types of risk factors that can evolve in burnout. It also emphasizes the relevance of the faculty resources in online education, as well as the link between resilience and burnout. On the one hand, resilience functions as a preventative measure against burnout. These findings offer intriguing suggestions for teachers, educators, and legislators in terms of classroom interventions that might help professors decrease burnout. First, our findings have implications for improving the teacher–student relationship, because a happy educator is a mentally healthy professor. Second, a faculty professor who is content with his or her supervisory relationship is more confident in his or her talents and better prepared to deal with academic challenges. As a result, supervisors should begin paying attention not only to the purely theoretical aspect of teaching enabling, but also to making a greater effort to connect with professors and lecturers, giving weight and attention to their difficulties, and fostering a classroom climate more devoted to sharing the problematic aspects of online teaching from the start of the academic year.

Furthermore, concentrating on the synergetic empowerment of both resilience and classroom connections, with particular attention to professor–student interactions, might be an effective way to work on burnout avoidance. In practice, principals and instructors should add periodic extra-curricular activities with the class group to the normal class hours, based on our findings, to strengthen the interaction between students and professors. Furthermore, and in line with previous statements, the university board could formally establish moments of exchange between lecturers and professors online, coordinated and monitored by supervisors, to share and exchange both the difficulties encountered in teaching and the most effective coping strategies used to deal with them. Creating a synergy between the class and the instructors brings up the prospect of constructing a class that functions as a resilient community where motivational and emotional issues are handled on a regular basis, and the resilient features of professors are emphasized.

### 5.1. Limitations to This Research

Surprising results were given when numerous professors were answering so quickly the questionnaire. They felt scared and alone copying with the pandemics. One limitation was to have carried out a transversal study in one moment of time only, not being able to compare the evolution of burnout and resilience at different moments of this pandemics.

### 5.2. Implications and Opportunities for Further Research

The current context of the Pandemics of COVID-19 opens several areas of research focused on adaptative mechanisms that professors are learning. It is important to compare resilience and burnout that professors have developed as part of their adaptation and how these variables impact in their copying mechanisms and their general response to this Pandemics. It would be important to compare the epidemiological differences of the pandemic between Mexico, North America and Europe, Asia, Africa and Australia. to evaluate if the potential differences affected the burnout within the Academic stuff.

In addition, a second application of the instrument is recommended to compare the evolution of these variables thru time. Facing COVID-19 pandemic shows the need for new conceptual and methodological research This opens up new opportunities for research that must be analyzed in the face of potential future pandemics and natural disasters that force organizations to be prepared and flexible in order to continue their operations and sustainability despite the situation. The objective of this study was achieved as far as determining the influence of resilience vs. burnout in Faculty professors.

Furthermore, the sample chosen represents a highly restricted group, making it impossible to extrapolate the findings to instructors at other levels (secondary) or in different subjects (private schools). Comparative approaches with other autonomous communities, intercultural or international studies, and other variables are now available as a result of the study. However, the findings reported may have practical implications for the benefit of faculty professors and lecturers.

## Figures and Tables

**Table 1:** Expected influence of the independent variable Resilience (exogenous) in connection with the dependent variables (endogenous).

Hypothesis	Exogenous Variable	Influence	Expected Sign	Endogenous Variables
H1	E14 Resilience	====>>	-	E9 Guilt
H2	E14 Resilience	====>>	-	E7 Mental Exhaustion
H3	E14 Resilience	====>>	-	E8 Indolence
H4	E14 Resilience	====>>	+	E6 Work excitement

Source: own elaboration.

**Table 2:** Demographics of the participants/Faculty members.

Gender	Marital Status	Age	Children	University Position	Scholarity Status	More than One Job	Time for Retirement
Women: 54%	Married: 53.3%	Older than 52 years: 20.5%	More than one child: 42%	adjunct professor: 63.1%	studying for a post grade: 20.1%	professors with more than one job: 62.5%	ten or more years: 81.9%
Men: 46%	Single: 25.8%	38–52 years: 45.1%	No children: 36.9%	full-time professor: 29.7%	Not studying: 79.9%	Only one job professors: 37.5%	Six years: 6.5%
	Other: 20.9%	Younger than 37 years: 34.4%	One child: 21.1%	lecturer: 7.2%			Less than 3 years: 11.6%

Source: Self research.

**Table 3:** Descriptive analysis: Differences between men and women.

	E6 Work Excitement	E7 Mental Exhaustion	E8 Indolence	E9 Guilt	E14 Resilience
Women	Mean = 4.42, Standard deviation = 0.753, T = −0.386, d.f. = 829, *p* = 0.699	Mean = 2.92, Standard deviation = 1.16, T = 6.313, d.f. = 829, *p* = 0.00	mean= 2.82, Standard deviation = 1.18, T = 6.231, d.f. = 829, *p* = 0.00	mean = 1.32, Standard deviation = 0.621 T = −2.165, d.f. = 829, *p* = 0.31	mean = 4.32, Standard deviation = 0.675 T = −2.205, d.f. = 829, *p* = 0.028
Men	E6 work excitement: mean: 4.44, Standard deviation = 0.74, T = 6.313, d.f. = 829, *p* = 0.00	E7 mental exhaustion: mean: 2.42, Standard deviation = 1.11, T = 6.313, d.f. = 829, *p* = 0.00	E8 indolence: mean: 2.31, Standard deviation = 1.13 T = 6.231, d.f. = 829, *p* = 0.00	E9 guilt: mean = 1.42, Standard deviation = 0.735, T = −2.165, d.f. = 829, *p* = 0.31	E14_resilience: mean = 4.42, Standard deviation = 0.624 T = −2.205, d.f. = 829, *p* = 0.028

Source: Self research.

**Table 4:** Descriptive statistics, reliability and validity of instruments.

Subscales	Mean	Standard Deviation	Cronbach’s Alpha	Rho_A	CR	AVE	R Squared	E14	E6	E7	E8	E9
E14_Resilience	4.37	0.65	0.89	0.89	0.91	0.56	- -	0.751				
E6_Work_excitement	4.44	0.75	0.91	0.92	0.94	0.74	0.238	0.450 **	0.862			
E7_Mental_exhaustion	2.69	1.17	0.93	0.95	0.95	0.83	0.102	−0.318 **	−0.269 **	0.91		
E8_Indolence	2.59	1.19	0.83	0.84	0.88	0.66	0.078	−0.332 **	−0.289 **	0.987 **	0.811	
E9_Guilt	1.37	0.68	0.92	0.94	0.94	0.77	0.057	−0.234 **	−0.175 **	0.244 **	0.256 **	0.875

** Significant correlation to level 0.01 (two-tailed). *n* = 831; CR = Composite reliability; AVE = Average variance extracted; Main diagonal = square root of AVE. Source: Self research.

**Table 5:** Bootstrapping of the final structural equations modeling.

Hypothesis	Subscales	Original (O) Sample	Mean (M) of the Sample	Standard Deviation (Std Dev)	T Statistics (|O/Std Dev|)	*p* Values
H1	E14_Resilience -> E6_Work_excitement	0.479	0.501	0.038	13.139	0
H2	E14_Resilience -> E7_Mental_exhaustion	−0.301	−0.298	0.035	8.537	0
H3	E14_Resilience -> E8_Indolence	0.279	−0.28	0.036	7.673	0
H4	E14_Resilience -> E9_Guilt	−0.245	0.247	0.042	5.864	0

Source: Self research.

**Table 6:** Hypotheses results findings.

Hypothesis	Result	Comments
H1	E14 resilience had an inverse, significative influence on E9 guilt with a std. beta of −0.238. This result shows a very low influence that explains only 1% of its variance from R square.	The low influence that resilience had on guilt do not diminish the importance of this relationship. In the sample, age group of older than 52 men, showed a higher manifestation of guilt; however, in this group resilience showed to be lower, an aspect that may be explained by the fact that elder women have stronger coping mechanisms against adversities at the workplace. This is coincident with another research [69,70,71,72,75,76,77,78].
H2	E14 resilience had an inverse, significative influence on E7 mental exhaustion with a std. beta of −0.32. This result explains only about 10% of its variance from R square	This result shows that resilience has an important role on mental exhaustion. However, in the sample, married professors with children had a higher resilient protection against mental exhaustion than the rest. this is related to the emotional support received from the family. This is coincident with another research [79,80].
H3	E14 resilience had an inverse, significative influence on E8 indolence with a std. beta of −0.279. This result explains only about 1% of its variance from R square	The low influence that resilience had on indolence do not diminish the importance of this relationship, we still can affirm that higher resilience reduces indolence, however, the sample group that had a higher indolence rate were women. This fact can be explained by their feeling of lack of support from male supervisors, inadequacy of schedules, excessive bureaucracy and the paperwork that results from it, and other factors, when combined with individual resources, which have a detrimental impact. The findings in this dimension corroborate the findings of the authors’ research [82,84].
H4	E14 Resilience had a direct, significative influence on E6 work excitement with a std. beta of −0.238. This result explains about 20% of its variance from R square	Resilience has a high influence in work excitement.There were no significant differences in E6 work excitement between men and women, positive factors of the job, such as reachable goals, empowerment, autonomy, organizational support, resource availability, and so on, increase professors perceived effectiveness, allowing him to control the demands and be conscious of his own abilities. The findings in this dimension corroborate the findings of the authors’ research [84,85,86].

Source: Self research.

## Data Availability

The data presented in this study are available upon request.

## Appendix A. Factor Loading of the Items

Subscales of factors: E14 Resilience; E6 Work excitement; E7 Mental exhaustion: E8 Indolence; E9 Guilt.

**Items**	**E14**	**E6**	**E7**	**E8**	**E9**
Q53R23	0.776				
Q53R21	0.773				
Q53R24	0.763				
Q53R17	0.761				
Q53R22	0.753				
Q53R20	0.751				
Q53R11	0.719				
Q53R5	0.706				
Q35R3		0.896			
Q35R4		0.895			
Q35R5		0.891			
Q35R2		0.871			
Q35R1		0.746			
Q36R2			0.939		
Q36R3			0.921		
Q36R4			0.905		
Q36R1			0.876		
Q37R1				0.846	
Q37R2				0.838	
Q37R4				0.823	
Q37R5				0.732	
Q38R3					0.918
Q38R2					0.907
Q38R5					0.887
Q38R4					0.865
Q38R1					0.794
